# Genetic Dysruption of the Histaminergic Pathways: A Novel Deletion at the 15q21.2 *locus* Associated with Variable Expressivity of Neuropsychiatric Disorders

**DOI:** 10.3390/genes13101685

**Published:** 2022-09-20

**Authors:** Carla Lintas, Roberto Sacco, Alessia Azzarà, Ilaria Cassano, Luigi Laino, Paola Grammatico, Fiorella Gurrieri

**Affiliations:** 1 Laboratory of Medical Genetics, Medical Genetics Unit, Department of Medicine, Università Campus Bio-Medico di Roma, Via Alvaro del Portillo 21, 00128 Rome, Italy; 2 Fondazione Policlinico Universitario Campus Bio-Medico di Roma, 00128 Rome, Italy; 3 UOC Genetica Medica, Azienda Ospedaliera S. Camillo-Forlanini, 00152 Rome, Italy

**Keywords:** histaminergic system, neuropsychiatric disorders, HDC gene, comorbidity

## Abstract

The involvement of the Histaminergic System (HS) in neuropsychiatric disease is not well-documented, and few studies have described patients affected by different neuropsychiatric conditions harbouring disruptions in genes involved in the HS. In humans, histamine is synthetised from histidine by the histidine decarboxylase enzyme encoded by the *HDC* gene (OMIM*142704). This is the sole enzyme in our organism able to synthetise histamine from histidine. Histamine is also contained in many different food types. We hereby describe a twenty-one-year-old female diagnosed with a borderline intellectual disability with autistic traits and other peculiar neuropsychological features carrying a 175-Kb interstitial deletion on chromosome 15q21.2. The deletion was inherited from the mother, who was affected by a severe anxiety disorder. The deleted region contains entirely the *HDC* and the *SLC27A2* genes and partially the *ATP8B4* gene. The *HDC* gene has been previously associated with Tourette Syndrome (TS). Based on the functional role of the *HDC*, we propose this gene as the best candidate to explain many traits associated with the clinical phenotype of our patient and of her mother.

## 1. Introduction

Neurodevelopmental disorders comprise a group of heterogeneous conditions, including autism spectrum disorder (ASD), intellectual disability, attention deficit hyperactivity disorder and learning disorder, which arise during the first decade of life [1]. According to the current view, neurodevelopmental disorders are considered multifactorial diseases with a strong genetic component in which rare and common variants with variable levels of penetrance interact with each other and with environmental factors. Comorbidity with other neuropsychiatric disorders, such as anxiety disorder, epilepsy and Tourette Syndrome (TS), is also very common [2]. Indeed, the same pathogenetic variants are often shared by different neuropsychiatric conditions, demonstrating that a few neurophysiological pathways are commonly disrupted in these disorders [3]. One of the less-studied pathways involved in neuropsychiatric disorders is the Histamine System (HS). Histamine is a biogenic amine responsible for a few major physiological functions: it brings messages to the brain, triggers the release of stomach acid to help digestion and is released upon injury or allergic reaction as part of the immune response. Histamine is contained in many different types of food, and the main histamine-producing cells are mast cells, basophils, histaminergic neurons in the basal ganglia of the brain and enterochromaffin-like cells (ECL) in the stomach [4]. Within our body, histamine is synthesised from histidine by the histidine decarboxylase enzyme, which is encoded by the *HDC* gene on chromosome 15q21.2. In peripheral organs, HDC mRNA is expressed predominately in the stomach, lungs and gallbladder. However, HDC mRNA expression is the highest in the central nervous system. Here, histamine is synthesised by the histaminergic neurons located within the tuberomammillary nucleus of the hypothalamus whose projections innervate the spinal cord, the brain stem and telencephalic regions. In addition to the histaminergic neurons, mast cells and the microglia also express the *HDC* gene, contributing to raising the histamine level within the CNS, especially in response to a brain injury. Four histamine receptors are expressed throughout the CNS and are encoded by *HRH1*, *HRH2*, *HRH3* and *HRH4* genes. The binding of histamine to its receptors activates microglia, which express all four receptors. The *HRH3* gene is also expressed in the presynaptic element of neurons, where it modulates the release of histamine but also of other neurotransmitters such as glutamate, GABA and serotonin. A fourth gene, the *HNMT* gene, is involved in the HS: it encodes for a histamine N-methyltransferase, which inactivates histamine by the addition of a methyl group. While the *HNMT* gene and the genes encoding for histamine receptors (*HRH1*, *HRH2*, *HRH3* and *HRH4*) are broadly expressed in the brain regions, the expression of *HDC* within the CNS is confined to the hypothalamus.

Within the CNS, the HS mediates several physiological functions, including cognition, attention, stress, memory, regulation of the sleep–wake cycle, sensory and motor functions. Furthermore, recent evidence supports a role for the HS in neuroglia activation, cytokine production and migration [5]. The overactivation of microglia has been observed in post-mortem ASD brains, and all the other HS functions mentioned above are altered in most neuropsychiatric disorders. The altered expression of *HNMT, HRH1, HRH2, HRH3* and *HRH4* in the post-mortem ASD dorsolateral prefrontal cortex has been documented in two independent data sets [6]. Furthermore, alterations in many genes involved in histamine receptor signalling are shared by autism and Tourette Syndrome patients [7], demonstrating a common and important role of the HS in different neurodevelopmental disorders.

The aim of the present work is to describe a young woman affected by borderline intellectual disability with autistic traits carrying a deletion encompassing the *HDC* gene. The CNV was transmitted by the mother who was affected by a severe anxiety disorder. In addition to genetic testing, a thorough neuropsychological phenotype analysis was carried out. The emerged neuropsychological profile supported a causal role for the HDC variant in the aetiology of her disease condition. To our knowledge, this is the first report of a patient affected by intellectual disability and additional neuropsychiatric conditions with haploinsufficiency of the *HDC* gene. We also reviewed the current literature for genotype–phenotype correlations in animal models and human diseases with a disruption of the HS pathway.

## 2. Materials and Methods

### 2.1. Family Enrollment

The proband was a 22-year-old female referred to the Genetic Counseling Service from Neuropsychiatric Clinics.

### 2.2. Array CGH

Peripheral blood was drawn from the patient and her parents into EDTA-containing tubes. Genomic DNA was extracted using the QIAamp DNA blood mini kit (Qiagen, Valentia, CA, USA), and CGH arrays were performed using the Kit CytoSure 8X60K ISCA v2 [hg19] (OGT). We could not perform a RNA quantitative analysis, as the *HDC* is not expressed in the blood under physiological conditions.

### 2.3. Retrospective Study

We introduced the following key words in PubMed for each of the six genes belonging to the HS pathway (*HDC*, *HNMT*, *HRH1*, *HRH2*, *HRH3* and *HRH4*): (1) name of the gene, (2) name of the gene AND autism, (3) name of the gene AND neurodevelopment and (4) name of the gene AND intellectual disability. We also searched the clinical databases ClinVar (https://www.ncbi.nlm.nih.gov/clinvar/ (accessed on 1 July 2022)), Decipher (https://www.deciphergenomics.org/ (accessed on 1 July 2022)) and Simon Foundation Autism Research Initiative (https://www.sfari.org/ (accessed on 1 July 2022)) for CNV and SNV (Single-Nucleotide Variants) associated with the six HS-associated genes.

## 3. Results

### 3.1. Clinical Presentation of the Patient and Neuropsychological Profile

The patient was administered a complete battery of neuropsychological/cognitive tests and questionnaires in order to outline the clinical phenotype (Table 1). She was the first and only child of apparently healthy unrelated parents and was born prematurely at 29 weeks of gestation, showing a severe degree of cerebral suffering. The birthweight was 0,750 Kg and height was 31 cm. She was hospitalised in intensive care for about 4 months. At birth, an atrial and interventricular septal defect was detected, which required closure by device placement (amplatzer septal occluder 14 mm). The clinical history was characterised by bronchodysplasia for severe prematurity, medically treated migraine with aura and vascular malformation in the right lower limb; at 10 years, an epileptic seizure was suspected, but a subsequent EEG examination did not show a clear epileptogenic aetiology. A brain MRI showed post-hypoxic–ischemic lesions. At 15 years, she underwent surgery treatment for arteriovenous fistula. The post-operative course was marked by the appearance of crises likely associated with panic attacks with tears, fears, partial loss of reality check and transient amnesia with the subsequent onset of headaches. Cognitive and motor development were characterised by a delay in the acquisition of language and calculation/arithmetic skills (learning disorder), dyspraxia, impaired attention, deficit in self-regulation of emotional response with agitation when frustrated and deficits in social skills. The parents also reported behavioural difficulties, including a tendency to isolate from her peers, poor communication with socio-pragmatic deficits, insistence on sameness with complex patterns of routinised and ritualistic behaviours, restrictive interests and activities, stereotypes, involuntary movements such as motor tics, marked asthenia with excessive sleepiness during the day and gastrointestinal problems with constipation. Noise hypersensitivity was also reported. All symptoms and behavioural traits described above were present during childhood and persisted into adulthood. No dysmorphic features were noted during physical examinations.

The patient shows an intellectual profile that can be placed at the lower limits of the standard norms (WAIS-IV: global IQ = 75), with not a significant discrepancy between verbal abilities (VIQ = 84) and visuo-perceptive abilities (NVIQ = 90); both working memory and visual-motor processing speed were poor (WMIQ = 72; PSIQ = 72). Significant cognitive and motor slowness were generally noticeable. Visuo-constructional abilities were normal (Rey complex figure). Some dimensions of executive functions were found in the normal range (Wisconsin card sorting test and Stroop test), while others appeared poor (Trail-making test, Tower of London and phonological fluency). Additionally, specific deficits were found for arithmetic skills and mental calculus. She was clinically diagnosed with an autism spectrum disorder due to a lack of social and emotional reciprocity, poor social abilities, impairment in nonverbal communication, nonmeaningful eye contact, poor facial expression and reduced mimicry; furthermore, the patient showed lower than average ability for understanding how other people feel and responding appropriately (ADOS-2, ADI-R, Autism spectrum quotient, Empathy quotient and Gilliam Asperger disorder scale). Adaptive skills across the lifespan appeared significantly impaired, as described by the parents (ABAS-II), especially regarding self-care, social abilities, self-control, life at home, school skills and management of one’s living environment. A psychopathological assessment was also carried out: the MMPI-2 clinical profile highlighted a mildly depressed mood, social introversion, anxious traits, obsessive–compulsive behaviours, generalised distress and difficulty in interpreting reality, with a tendency to attribute personal meanings to neutral events (self-reference thoughts).

### 3.2. Genetic Findings

**Figure 1:**
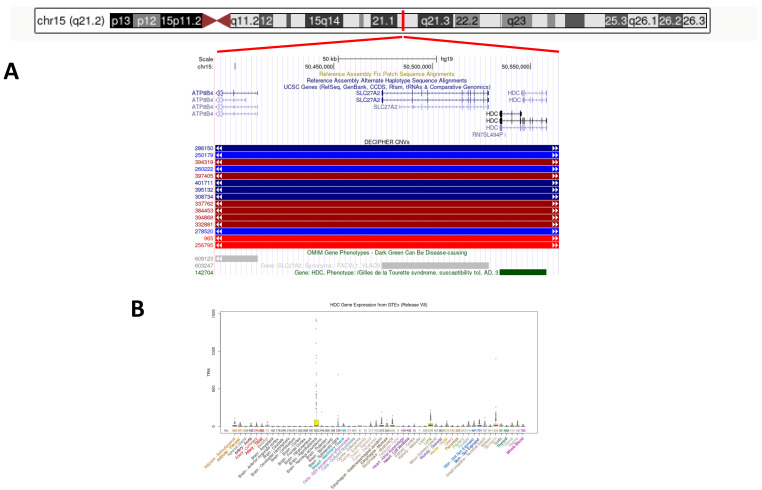
(**A**) Chromosomal location of the 15q21.2 deletion and its gene content. (**B)** Tissue expression profile of the HDC gene taken from the GTEx database.

The 15q21.2 deletion was detected by the array CGH (Kit CytoSure 8X60K ISCA v2, hg19) from 50,389,918 bp to 50, 564,328, encompassing a genomic region of approximately 175 Kb. The deletion was transmitted by the mother and involved entirely the *HDC* (OMIM*142704) and the *SLC27A2* (OMIM*603247) genes and partially the *ATP8B4* (OMIM*609123) (Figure 1). The *ATP8B4* gene encodes for an ATPase (Class I, type 8B), whereas the *SLC27A2* encodes for a fatty acid transporter. According to the GTEx database of gene expression (https://www.gtexportal.org/home/ (accessed on 1 July 2022)), all these genes are not significantly expressed in the blood, and therefore, we could not perform RNA expression studies. Within the deletion, the *HDC* gene appears to be the strongest candidate to explain our patient’s phenotype. The *HDC* gene is expressed at the highest level in the hypothalamus, lungs and stomach and to low levels in other tissues (Figure 1). The database Decipher (http://decipher.sanger.ac.uk/ (accessed on 1 July 2022)) reports eight patients with chr15q21.2 deletions fully overlapping the one detected in our proband. However, these deletions are much larger with respect to that of our patient, ranging in size from 8 Mb up to more than 10 Mb, and therefore, no comparisons can be done. No similar-sized deletions are reported in the Database of Genomic Variants of the general population (DGV: http://dgv.tcag.ca/dgv/app/home (accessed on 1 July 2022)), further supporting the pathogenetic role of the CNV found in the proband and in her mother (Figure 1).

## 4. Discussion

Genes involved in the HS have been occasionally found mutated in patients with neuropsychiatric disorders. A nonsense heterozygous mutation (p.Trp317Stop) in the *HDC* gene was identified in a two-generation pedigree: the father and eight children carrying the variant in heterozygosity were affected by Tourette Syndrome ([8] Table 2). In addition, the father and three children had obsessive compulsive disorder. The disease segregated in the family has an autosomal dominant trait, and the mutant truncated protein acted through a dominant negative mechanism. In line with this finding, Ohtsu and colleagues [9] demonstrated that the histamine concentration in the central nervous system of HDC-deficient mice was lower, and mice lacked histamine-synthesising activity. A case–control study with 529 European families affected by Tourette Syndrome demonstrated an over-transmission of two SNPs (rs 854150 and rs 1894236) located within the *HDC* genomic region, supporting the role for the HS in neurodevelopment [10]. The genotype of our patient is compatible with a decrease in the enzyme encoded by the *HDC* gene due to the loss of one allele. Indeed, she displays several characteristics found in KO mouse and patients with *HDC* mutations, such as jerky movements, constipation, excessive daytime sleepiness and emotional dysregulation. Furthermore, the anxiety disorder affecting her mother can also be related to HS dysregulation due to the loss of one copy of the *HDC* gene.

In addition to the *HDC* gene, homozygous mutations in the *HNMT* gene, involved in the deactivation of histamine, have also rarely been reported (Table 2). The *HNMT* enzyme is the sole enzyme responsible for the termination of histamine actions in the brain. Two different missense variants (Gly60Asp and Leu208Pro) and one nonsense variant (Glu30Stop) have been reported in three different families. The two missense variants were identified in two unrelated consanguineous pedigrees of Iranian and Kurdish ancestry, having four and three affected individuals, respectively. All individuals were affected by intellectual disability ranging from severe (6/7) to mild (1/7). In the Iranian family, females were more severely affected than males, whereas, in the Kurdish family, no sex difference was observed with respect to intellectual disability severity. Functional and in silico studies showed that the p. Gly60Asp variant disrupted the enzymatic activity of the protein, whereas the p.Leu208Pro variant was associated with a reduced protein stability, causing a decreased histamine inactivation ([11] Table 2). A more recent study ([12] Table 2) identified a *HNMT* homozygous stop mutation (p.Gln30Stop) in an adolescent male patient with severe intellectual disability from a consanguineous family. In addition to global regression around the age of four years, this patient had gastrointestinal problems, sleep disturbance, autistic disorder and aggressive behaviour. Treatment with the antihistaminergic compound hydroxyzine and a histamine-restricted diet resulted in the improvement of many symptoms, including the normalisation of sleep patterns, complete continence, speech improvement and reduction of aggressive behaviour.

Two additional studies ([13,14] Table 2) reported heterozygous deleted CNV encompassing the *HNMT* gene in patients affected by neurodevelopmental disorders. However, the CNVs were very large and encompassed many other genes that may also contribute to the phenotype.

Knockout (KO) mouse animal models have been engineered for both *HDC* and *HNMT* genes. *HDC* homozygous and heterozygote null mice both developed Tourette-like syndrome, and an injection with histamine significantly reduced the stereotypies [15]. *HDC* KO mice appear similar to TS patients with the *HDC* Trp317Stop variant and also have increased levels of dopamine and elevated dopamine receptors in the substantia nigra [16]. In addition, mice lacking *HDC* show a reduced number of mast cells with less granules and altered morphology [9]. They also display gastrointestinal problems such as indigestion, diarrhoea and constipation. These symptoms can be related to the lack of acidification by gastric juice, whose synthesis is normally triggered by histamine.

*HNMT* KO mice exhibit aggressive behaviour and dysregulation of the sleep–wake cycle, characterised by prolonged awakening during the light and more sleepiness during the night. Additionally, the histamine levels were six-fold higher in the KO mice compared to the wild-type mice and led to impaired cognitive and behavioural phenotypes.

Overall, these observations suggest that the fine tuning of histamine levels in the brain is crucial for behavioural balance and neuropsychological development. Low histamine levels likely contributed to the complex neuropsychological phenotype of our patient and of her mother, both carrying a deletion encompassing the entire *HDC* gene. The observed genetic loss is compatible with a dysregulation of the HS system and can explain many phenotypic traits of the proband as jerky movements, constipation, excessive daytime sleepiness and emotional dysregulation, which resemble, in part, those observed in null mice models for HDC. Since the proband is more severely affected than her mother, we cannot exclude the contribution of the perinatal injury to her phenotype. A diet based on histamine-rich products should be considered to see if the symptoms improve.

## Figures and Tables

**Table 1:** Neuropsychological test results.

Cognitive Test	Score or Level of Performance
WAIS-IV	
- Verbal comprehension IQ - Visuo-perceptual reasoning IQ - Working memory IQ - Visuo-motor speed IQ - Full scale IQ	84 (normal) 90 (normal) 72 (poor) 72 (poor) 75 (poor)
Executive functions	
- Trail making test - Stroop test - Wisconsin card sorting test/set-shifting ability - Tower of London - Phonological fluency	Poor Normal Normal Poor Poor
Visuo-constructional abilities	
- Rey complex figure	Normal
Arithmetical abilities	
- Mental calculus - Mathematical reasoning	Poor Poor
ADOS-2	
- Language and communication	1
- Social interaction	7
Total score	8 (ASD threshold = 7)

**Table 2:** Genotype–phenotype correlations relative to variants found in *HDC* and *HNMT* genes.

Gene	Function	CNV/SNV	Phenotype	Additional Phenotypic Features	Ref.
HDC	Conversion of histidine into histamine	p.Trp317Stop (heterozygous)	Tourette syndrome (two-generation pedigree)	Two children and their father had also obsessive compulsive disorder	[8]
		Family study rs854150 and rs1894236 520 European trios	Tourette syndrome	Attention deficit Hyperactivity Disorder, obsessive compulsive disorder	[10]
		Inherited CNV (loss, heterozygous) chr15:50,389,918-50,564,328 (hg19)	Mild intellectual disability	Emotional dysregulation, excessive daytime sleepness, constipation, autistic traits, dyspraxia, learning disorder, sleep disturbance, pervasive and perservant behaviour	This study
HNMT	Inactivation of histamine	de novo CNV (loss, heterozygous) chr2:137,234,086–144,547,896 (hg18)	Autism	-	[13]
		de novo CNV (loss, heterozygous) chr2:138,750,000-144,750,000 (hg19)	Severe intellectual disability, autism, essential hypertension, congenital malformations	-	[14]
		p.Gly60Asp (homozygous) p.Leu208Pro (homozygous)	Intellectual disability ranging from mild to severe in two not related pedigrees	Delayed speech development in the Iranian pedigree and mild regression from the age of 5 years in the Kurdish pedigree. No dysmorphisms	[11]
		p.Gln30Stop(homozygous)	Severe intellectual disability	Autism, aggression, sleep disturbance, global regression at the age of 4 years, delayed speech, gastrointestinal problems. No dysmorphisms	[12]

## Data Availability

The research data were not shared.
